# Clinically Relevant Extended-Spectrum β-Lactamase–Producing *Escherichia coli* Isolates From Food Animals in South Korea

**DOI:** 10.3389/fmicb.2020.00604

**Published:** 2020-04-22

**Authors:** Jihyun Song, Sung-Suck Oh, Junghee Kim, Sukyoung Park, Jinwook Shin

**Affiliations:** ^1^Department of Microbiology, College of Medicine, Inha University, Incheon, South Korea; ^2^Incheon Research Institute of Public Health and Environment, Incheon, South Korea

**Keywords:** *Escherichia coli*, extended-spectrum β-lactamase, multidrug resistance, food animal, South Korea

## Abstract

Extended-spectrum β-lactam antimicrobials have been broadly used in food animals and humans to control infectious diseases. However, the emergence and rapid spread of extended-spectrum β-lactamase (ESBL)–producing Enterobacteriaceae, mainly *Escherichia coli*, have seriously threatened global health in recent decades. In this study, we determined the prevalence, antimicrobial susceptibility, and genetic properties of ESBL-producing *E. coli* (ESBL-EC) strains isolated from food animals in South Korea. A total of 150 fecal samples from healthy chickens (*n* = 34), pigs (*n* = 59), and cattle (*n* = 57) were screened from January to July 2018. Among these, 77 non-duplicate cefotaxime-resistant ESBL-EC strains were isolated from 32 chicken, 41 pig, and 4 cattle samples, with the corresponding occurrence rates of 94.1, 69.5, and 7.0%, respectively. All the isolates showed multidrug resistance (MDR) and produced at least one type of β-lactamase, including CTX-M (98.7%) and TEM (40.3%). CTX-M-14 (53.1%), CTX-M-55 (53.7%), and CTX-M-65 (50.0%) were the predominant genotypes in the chicken, pig, and cattle samples, respectively. Multilocus sequence typing revealed 46 different sequence types (STs), including the human-associated extraintestinal pathogenic *E. coli* ST131 (*n* = 2), ST10 (*n* = 5), ST38 (*n* = 1), ST410 (*n* = 4), ST354 (*n* = 2), ST58 (*n* = 3), ST117 (*n* = 1), and ST457 (*n* = 1). To the best of our knowledge, this is the first report of pandemic *E. coli* ST131 in non-human isolates in South Korea. Our results demonstrate the high prevalence and diversity of MDR-ESBL-EC in food animals and highlight them as potential pathogenic ESBL-EC reservoirs that may pose a high risk to human health.

## Introduction

Extended-spectrum β-lactam antimicrobials have been widely used to treat bacterial infections both in humans and animals. Since the first extended-spectrum β-lactamase (ESBL) was described in Germany in 1983, the global spread of ESBL-producing *Escherichia coli* (ESBL-EC), including the pandemic *E. coli* sequence type (ST) 131 clone, has led to a rapid increase in the population of ESBL-EC strains worldwide (Pitout and Laupland, 2008; Rogers et al., 2011). The most widespread ESBLs are CTX-M–type β-lactamases, which can be divided into five major groups (CTX-M groups 1, 2, 8, 9, and 25) (Bonnet, 2004; Canton et al., 2012; Bevan et al., 2017), and at least 214 CTX-M variants have been detected^1^ accessed January 24, 2020. Among these, CTX-M-15 in the CTX-M group 1 and CTX-M-14 in the CTX-M group 9 are prevalent in most countries (Bevan et al., 2017). Likewise, both variants have been predominantly detected in clinical ESBL-EC isolates in South Korea (Song et al., 2009; Kim et al., 2019).

As ESBL-EC strains are rising in humans, they have also been increasingly isolated from food animals in different geographical regions, including China (Rao et al., 2014), Germany (Laube et al., 2013), Netherlands (Hordijk et al., 2013), Tunisia (Maamar et al., 2016), and United States (Markland et al., 2019). Moreover, multidrug-resistant (MDR) ESBL-EC pathogens, which pose a serious threat to human health due to the limited treatment options, extensively disseminate among food animals (Ho et al., 2011; Vitas et al., 2018), which are considered to be the primary reservoirs of antimicrobial-resistant enteric bacteria, although the routes of transmission to humans are unclear. Such bacteria can presumably pass through the food chain or via close contact and can colonize the intestines of humans (Carattoli, 2008). In fact, the same genetic elements and/or STs have been observed between human and food animal isolates of ESBL-EC (Moodley and Guardabassi, 2009; Leverstein-van Hall et al., 2011; Tamang et al., 2013a; Hammerum et al., 2014; Dahms et al., 2015), suggesting the possibility of clonal and genetic transmissions between these settings. Previous studies conducted in South Korea have mainly focused on the prevalence and characteristics of ESBL genes of *E. coli* isolates from food animals (Tamang et al., 2013b; Shin et al., 2017), but their relatedness to human-associated clonal lineages has rarely been investigated.

In this study, we evaluated the prevalence, antimicrobial susceptibility, and molecular genetic features of ESBL-EC strains isolated from food animals in South Korea. Furthermore, we assessed the epidemiological relatedness of the clonal populations to human-associated *E. coli* STs according to a national surveillance program.

## Materials and Methods

### Isolation and Identification of ESBL-EC From Food Animals

A total of 150 healthy food animals, including 34 chickens, 59 pigs, and 57 cattle, were obtained from 28, 34, and 53 farms (115 in total), respectively, across the country in South Korea. Fecal samples were collected from the intestinal tracts of individual animals slaughtered at the slaughterhouses. For *E. coli* isolation, 0.1 g of the samples was inoculated to 9 mL of Tryptone Soya Broth (Oxoid, Basingstoke, United Kingdom) containing 0.4 μg/mL vancomycin (Wako Pure Chemical Industries, Hyogo, Japan) and incubated at 37°C for 4 h. A loopful of each enrichment was streaked on MacConkey screening plate supplemented with 2 μg/mL cefotaxime and incubated at 37°C for 24 h. Subsequently, one pink or reddish colony suspected of comprising *E. coli* from each fecal sample was randomly selected using a sterile platinum loop and cultured on CHROMagar ESBL (CHROMagar, Paris, France) at 37°C for 24 h. One dark pink to reddish single colony selected from each plate was grown on Tryptone Soya Agar (Oxoid) at 37°C for 4 h, and the pure isolates were used for further characterization. The species of the isolates were identified using matrix-assisted laser desorption ionization–time of flight mass spectrometry (Bruker Daltonik GmbH, Bremen, Germany) with score values ≥2.0. Extended-spectrum β-lactamase production was confirmed via the double-disk synergy test using disks containing amoxicillin–clavulanic acid (20/10 μg), cefotaxime (30 μg), cefepime (30 μg), and ceftazidime (30 μg).

### Antimicrobial Susceptibility Testing

Antimicrobial susceptibilities testing was performed by the disk diffusion method in accordance with the guidelines of the Clinical and Laboratory Standards Institute (CLSI, M100-S27) (CLSI, 2017) using commercial disks (Oxoid) supplemented with 21 antimicrobial agents as follows: gentamicin (10 μg), amikacin (30 μg), ertapenem (10 μg), imipenem (10 μg), meropenem (10 μg), cefazolin (30 μg), cefotaxime (30 μg), ceftazidime (30 μg), cefepime (30 μg), cefoxitin (30 μg), ciprofloxacin (5 μg), nalidixic acid (30 μg), trimethoprim-sulfamethoxazole (1.25/23.75 μg), tigecycline (15 μg), aztreonam (30 μg), ampicillin (10 μg), piperacillin (100 μg), amoxicillin–clavulanic acid (20/10 μg), ampicillin–sulbactam (10/10 μg), chloramphenicol (30 μg), and tetracycline (30 μg). Mueller–Hinton agar plate (Difco Laboratories, Detroit, MI, United States) was swabbed with a pure ESBL-EC suspension adjusted to a 0.5 McFarland standard. The disks were placed on the agar using a disk dispenser (Oxoid), and the plate was incubated at 37°C for 24 h. The zone diameters of growth inhibition were measured using an electronic caliper. All of the results were interpreted according to the zone diameter breakpoints of the CLSI guidelines (CLSI, 2017) except that the tigecycline results were interpreted according to the European Committee for Antimicrobial Susceptibility Testing (EUCAST) breakpoint version 7.1 (EUCAST, 2017; Supplementary Table S1). *Escherichia coli* ATCC 25922 was used as the reference strain. Multidrug resistance was determined as non-susceptibility to at least one agent in ≥3 antimicrobial classes, and extensive drug resistance (XDR) was defined as non-susceptibility to at least one agent in all but ≤2 classes (Magiorakos et al., 2012).

### Molecular Characterization of ESBL Genes

Total DNAs of ESBL-EC isolates were extracted and purified using G-spin Total DNA Extraction Kit (iNtRON Biotechnology, Seongnam, South Korea) according to the manufacturer’s instructions. To identify the ESBL alleles of the isolates, the extracts were subjected to polymerase chain reaction (PCR) analyses using the specific primer pairs, including *bla*_TEM_, *bla*_SHV_, and *bla*_CTX–M_ groups 1, 2, 9, and 25 (Supplementary Table S2). After the bidirectional Sanger sequencing of the amplicons by BIOFACT (Daejeon, South Korea), the resultant sequences were compared with the published β-lactamase gene sequences from the GenBank database of the NCBI using the online BLAST program^2^.

### Conjugation and Plasmid Typing Assay

Conjugation assay was performed with 24 *bla*_CTX–M–55_-positive donors isolated from food animals and *E. coli* J53-Azi^R^ recipient strains. Transconjugants were selected on MacConkey agar plates containing 2 μg/mL of cefotaxime and 100 μg/mL of sodium azide. The presence of the *bla*_CTX–M–55_ gene in the transconjugants was confirmed by PCR analysis using the CTX-M-1F/R primer pair described in Supplementary Table S2. Replicon typing of the transconjugant plasmids were tested for the major plasmid incompatibility groups among Enterobacteriaceae (HI1, HI2, I1-Iγ, X, L/M, N, FIA, FIB, W, Y, P, FIC, A/C, T, FIIA_S_, F, K, and B/O) using a PCR-based replicon typing method (Carattoli et al., 2005). The IncF plasmids containing FIB were further analyzed for distinguishing variants by a replicon sequence typing (RST) scheme (Supplementary Table S2) as previously described (Villa et al., 2010). The results of sequences were compared with the plasmid multilocus sequence typing (MLST) database deposited at http://pubmlst.org/plasmid/. The number of plasmids in the transconjugants was solved by pulsed field gel electrophoresis (PFGE) with S1 nuclease (Thermo Fisher Scientific, Waltham, MA, United States) digestion of total DNA using the CHEF MAPPER system (Bio-Rad Laboratories, Hercules, CA, United States).

### Phylogenetic Characterization

The DNA extracts of the isolates were used for multiplex PCR targeting *chuA*, *yjaA*, and the DNA fragment TspE4.C2, as described previously (Clermont et al., 2000). Phylogroups A and B1 are typically commensal, whereas groups B2 and D are extraintestinal virulence-associated strains (Johnson et al., 2001). The ST genotypes of the isolates were determined by PCR-based sequencing of the housekeeping genes (*adk*, *fumC*, *gyrB*, *icd*, *mdh*, *purA*, and *recA*) followed by MLST in accordance with the Enterobase protocol and database^3^ (Wirth et al., 2006). The sequences of seven housekeeping genes were aligned using Clustal W, and the phylogenetic tree was constructed using MEGA version X based on the maximum-likelihood method using Kimura’s two-parameter model with gamma distribution and invariant sites. The *E. coli* phylogeny was estimated by a bootstrap analysis with 1,000 replicates (Kumar et al., 2018).

## Results

### Prevalence and Antimicrobial Susceptibility of ESBL-EC Strains in Food Animals

**FIGURE 1:**
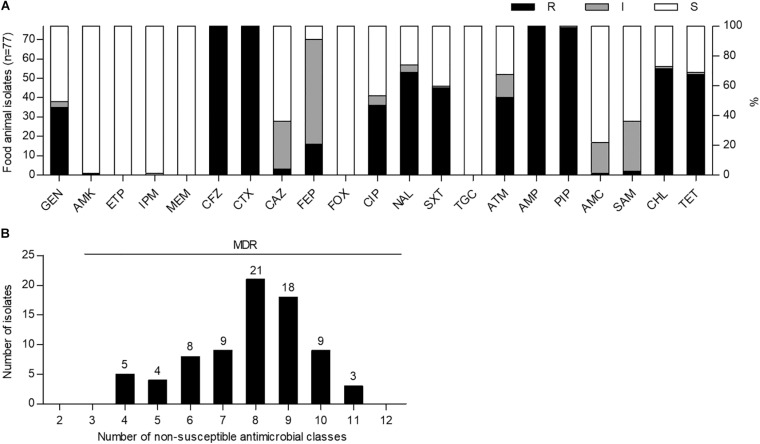
Antimicrobial susceptibility and multidrug resistance profiles of cefotaxime-resistant *Escherichia coli* isolates from food animals in South Korea. **(A)** Antimicrobial susceptibilities were analyzed by the disk agar diffusion method. GEN, gentamicin; AMK, amikacin; ETP, ertapenem; IPM, imipenem; MEM, meropenem; CFZ, cefazolin; CTX, cefotaxime; CAZ, ceftazidime; FEP, cefepime; FOX, cefoxitin; CIP, ciprofloxacin; NAL, nalidixic acid; SXT, trimethoprim-sulfamethoxazole; TGC, tigecycline; ATM, aztreonam; AMP, ampicillin; PIP, piperacillin; AMC, amoxicillin–clavulanic acid; SAM, ampicillin–sulbactam; CHL, chloramphenicol; TET, tetracycline; R, resistant; I, intermediate resistant; S, susceptible. **(B)** Multidrug resistance was determined as non-susceptibility to at least one agent in three or more antimicrobial classes. MDR, multidrug resistance.

To investigate the prevalence of ESBL-EC strains in food animals, we cultured a total of 150 fecal samples from 34 chickens, 59 pigs, and 57 cattle on MacConkey agar plates containing cefotaxime (2 μg/mL) and then purified single colonies on CHROMagar ESBL plates. Among them, 77 non-duplicate cefotaxime-resistant *E. coli* strains were isolated from 32 chickens (94.1%), 41 pigs (69.5%), and 4 cattle (7.0%). Antimicrobial susceptibility testing of 21 antimicrobial substances from 14 classes showed that all of the isolates were not susceptible to cefotaxime (extended-spectrum cephalosporin class), cefazolin (non-extended-spectrum cephalosporin class), and ampicillin and piperacillin (penicillin class), indicating MDR (≥4 classes) phenotypes; however, they were susceptible to ertapenem, meropenem, cefoxitin, and tigecycline (Figure 1 and Supplementary Table S3). High frequencies of amikacin (76/77, 98.7%) and imipenem (76/77, 98.7%) susceptibilities were also observed. Although there was no XDR strain, three pig isolates (EC21, EC37, and EC43) showed intermediate resistance or resistance to at least one agent in all but three antimicrobial classes investigated, as described in Supplementary Tables S4, S5.

### Characterization of ESBL Genes

Next, the genotypes responsible for ESBL production were investigated using PCR-based sequencing for *bla*_TEM_, *bla*_SHV_, and *bla*_CTX–M_ groups 1, 2, 9, and 25. The most prevalent ESBL genotype was represented by *bla*_CTX–M_ group 1 (46/77, 59.7%), followed by *bla*_CTX–M_ group 9 (31/77, 40.3%) (Table 1). The sequence identities with the β-lactamase gene sequences of the NCBI database using BLAST search were as follows: *bla*_TEM–1_ (>99.5%), *bla*_CTX–M–1_ (>99.8%), *bla*_CTX–M–3_ (99.9%), *bla*_CTX–M–15_ (>99.4%), *bla*_CTX–M–55_ (>99.3%), *bla*_CTX–M–14_ (>99.7%), and *bla*_CTX–M–65_ (>99.6%). All sequenced *bla*_TEM_ amplicons belonged to non-ESBL *bla*_TEM–1_ (31/77, 40.3%), whereas *bla*_SHV_ and *bla*_CTX–M_ groups 2 and 25 were not detected. The predominant CTX-M–type ESBLs were CTX-M-14 in chickens (17/32, 53.1%), CTX-M-55 in pigs (22/41, 53.7%), and CTX-M-65 in cattle (2/4, 50.0%). CTX-M-1, CTX-M-3, and CTX-M-15 were also identified at various frequencies in each animal species. The ESBL gene profile of an individual isolate is given in Supplementary Table S4. In addition, we carried out the conjugation assay with azide-resistant *E. coli* J53 recipient strain, focusing on *bla*_CTX–M–55_. The transmission of *bla*_CTX–M–55_ gene was observed in 14 of 24 *bla*_CTX–M–55_-positive ESBL-EC isolates (Supplementary Table S6). The J53 transconjugants were further analyzed using PCR-based replicon typing. F, FIB, I1-Iγ, K, N, and FIA replicons were identified in 12, 10, 5, 3, 2, and 1 transconjugants (Supplementary Table S6). The RST discriminated F1 (9/10, 90.0%) and F20 (1/10, 10.0%) plasmids among the IncF plasmids containing FIB. S1-PFGE analysis revealed that 10 transconjugants (EC4, EC7, EC10, EC16, EC18, EC19, EC31, EC33, EC39, and EC62) contained one plasmid, whereas four (EC17, EC21, EC35, and EC40) contained two plasmids.

**TABLE 1:** Extended-spectrum β-lactamase genotypes of cefotaxime-resistant *Escherichia coli* isolates from food animals.

***bla* genotype**	**Number (%) of cefotaxime-resistant** ***E. coli* isolates**			
	**Chicken**	**Pig**	**Cattle**	**Total**
	**(*n* = 32)**	**(*n* = 41)**	**(*n* = 4)**	**(*n* = 77)**
TEM-1	10 (31.3)	21 (51.2)	0	31 (40.3)
CTX-M groups	32 (100)	40 (97.6)	4 (100)	76 (98.7)
CTX-M group 1	10 (31.3)	33 (80.5)	2 (50.0)	45 (58.4)
CTX-M group 9	21 (65.6)	7 (17.1)	2 (50.0)	30 (39.0)
CTX-M group 1 and group 9	1 (3.1)	0	0	1 (1.3)
CTX-M genotypes	5 (15.6)			
CTX-M-1		0	0	5 (6.5)
CTX-M-3	0	1 (2.4)	0	1 (1.3)
CTX-M-15	4 (12.5)	10 (24.4)	1 (25.0)	15 (19.5)
CTX-M-55	1 (3.1)	22 (53.7)	1 (25.0)	24 (31.2)
CTX-M-14	16 (50.0)	4 (9.8)	0	20 (26.0)
CTX-M-65	5 (15.6)	3 (7.3)	2 (50.0)	10 (13.0)
CTX-M-15 and CTX-M-14	1 (3.1)	0	0	1 (1.3)

### Phylogenetic Analysis of ESBL-EC Isolates

**FIGURE 2:**
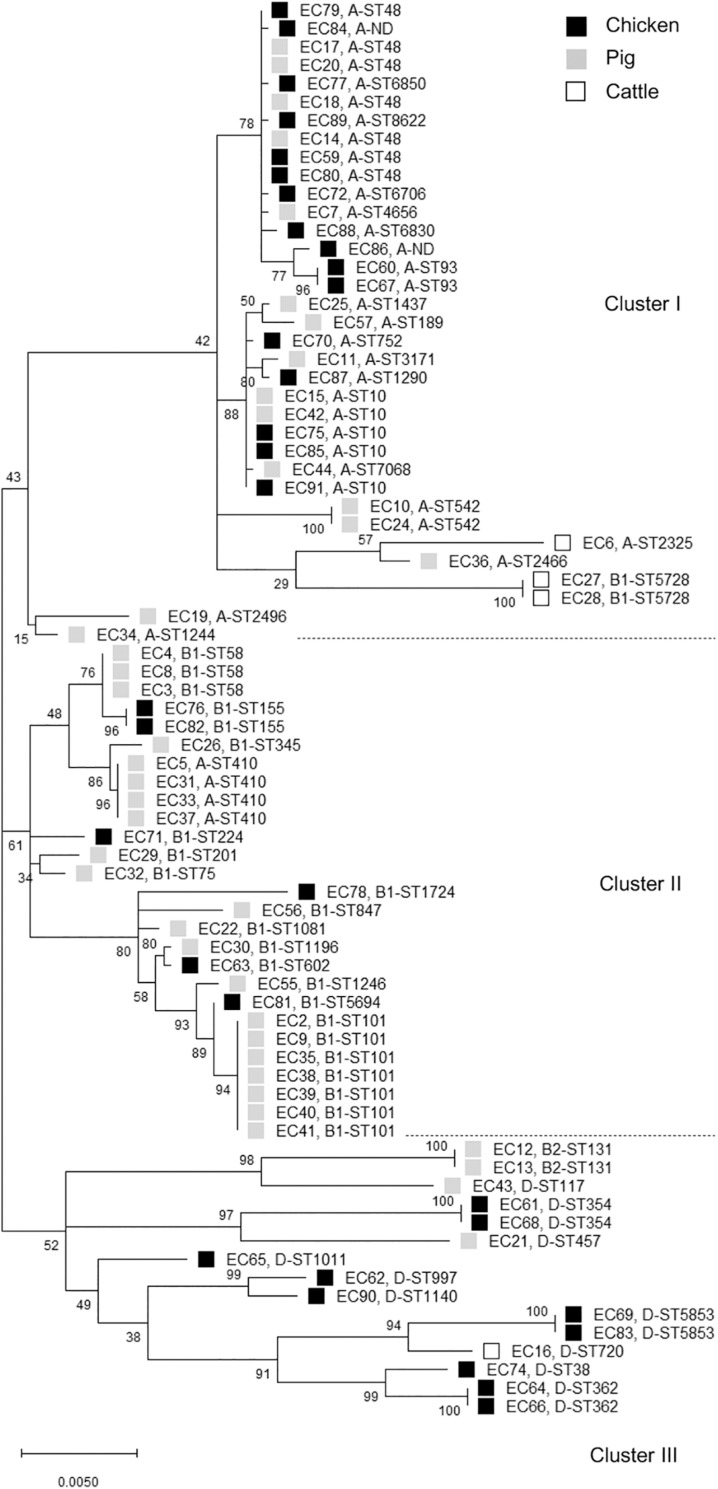
Phylogenetic tree of cefotaxime-resistant *Escherichia coli* isolates from chickens, pigs, and cattle. The maximum likelihood phylogenetic tree was constructed using Mega X software based on the seven housekeeping genes (*adk*, *fumC*, *gyrB*, *icd*, *mdh*, *purA*, and *recA*) and Kimura’s 2-parameter model. Bootstrap support percentages (1,000 replicates) were indicated in the different branches. Scale bar at the bottom represents the genetic distance. The phylogroup and sequence type (ST) of each isolate were displayed. Black, gray, and white squares represent chicken, pig, and cattle, respectively. ND, not determined ST.

The distribution of phylogroups showed that subgroup A was predominant (37/77, 48.1%), followed by subgroups B1 (25/77, 32.5%), D (13/77, 16.9%), and B2 (2/77, 2.6%) in ESBL-EC isolates from food animals (Table 2), thus suggesting the higher distribution of commensal groups A and B1 than pathogenic groups B2 and D. However, the subgroups were differentially distributed among the animal species. The chicken isolates mainly belonged to subgroups A and D, whereas the pig isolates mostly belonged to subgroups A and B1. Through MLST analysis of a total of 77 isolates, we determined 46 distinct *E. coli* STs (21 chickens, 24 pigs, and 3 cattle), among which ST10 and ST48 were found in both chicken and pig isolates, and 2 unknown STs (Table 3). The proportions of STs detected only once among the isolates were 43.8% (14/32) in chickens, 41.5% (17/41) in pigs, and 50.0% (2/4) in cattle. The highest number of chicken isolates belonged to ST10 or ST48 (3/32, 9.4% each). Among the pig isolates, ST101 was the most prevalent lineage (7/41, 17.1%) and pandemic ST131 was also identified (2/41, 4.9%). The MLST-based phylogenetic tree showed that ESBL-EC isolates were branched into three major clusters, among which Cluster I and Cluster II consisted of 89.2% of subgroup A and 92% of subgroup B1, respectively, and Cluster III included all of the subgroups B2 and D (Figure 2).

**TABLE 2:** Phylogroups of cefotaxime-resistant *Escherichia coli* isolates from food animals.

**Phylogroup**	**Number (%) of cefotaxime-resistant *E. coli* isolates**			
	**Chicken (*n* = 32)**	**Pig (*n* = 41)**	**Cattle (*n* = 4)**	**Total (*n* = 77)**
A	16 (50.0)	20 (48.8)	1 (25.0)	37 (48.1)
B1	6 (18.8)	17 (41.5)	2 (50.0)	25 (32.5)
B2	0	2 (4.9)	0	2 (2.6)
D	10 (31.3)	2 (4.9)	1 (25.0)	13 (16.9)

**TABLE 3:** MLST analysis of cefotaxime-resistant *Escherichia coli* isolates from food animals.

**MLST**	**Number (%) of cefotaxime-resistant** ***E. coli* isolates**			
	**Chicken**	**Pig**	**Cattle**	**Total**
	**(*n* = 32)**	**(*n* = 41)**	**(*n* = 4)**	**(*n* = 77)**
ST10	3 (9.4)	2 (4.9)	0	5 (6.5)
ST48	3 (9.4)	4 (9.8)	0	7 (9.1)
ST58	0	3 (7.3)	0	3 (3.9)
ST93	2 (6.3)	0	0	2 (2.6)
ST101	0	7 (17.1)	0	7 (9.1)
ST131	0	2 (4.9)	0	2 (2.6)
ST155	2 (6.3)	0	0	2 (2.6)
ST354	2 (6.3)	0	0	2 (2.6)
ST362	2 (6.3)	0	0	2 (2.6)
ST410	0	4 (9.8)	0	4 (5.2)
ST542	0	2 (4.9)	0	2 (2.6)
ST5728	0	0	2 (50.0)	2 (2.6)
ST5853	2 (6.3)	0	0	2 (2.6)
Once detected ST	14 (43.8)	17 (41.5)	2 (50.0)	33 (42.9)
Not determined ST	2 (6.3)	0	0	2 (2.6)

*ST, sequence type.*

## Discussion

The prevalence of ESBL-EC in food animals (94.1% in chickens, 69.5% in pigs, and 7.0% in cattle) in this study was found to be higher than those reported in previous investigations before and after 2010 (33.3% in chickens, 21.5% in pigs, and 0.2% in cattle) (Tamang et al., 2013b; Lim et al., 2015) in South Korea. In the susceptibility testing for 14 antimicrobial classes, all of the isolates showed the MDR phenotypes with a resistance range of 4–11 classes (Figure 1B). In general, ESBLs can hydrolyze extended-spectrum cephalosporins and monobactams but not carbapenems and cephamycins and are inhibited by β-lactamase inhibitors (Canton et al., 2012). Consistent with these properties, ESBL-EC isolates in this study showed a relatively high susceptibility to ertapenem, imipenem, meropenem, cefoxitin, amoxicillin − clavulanic acid, and ampicillin − sulbactam (Figure 1A and Supplementary Table S3). In addition, ESBL-producing Enterobacteriaceae among clinical isolates have been reported to be mostly susceptible to tigecycline and amikacin (Morosini et al., 2006; Denisuik et al., 2019), and similarly, the same phenotype was also found in food animal isolates in this study (Figure 1A and Supplementary Table S3).

The epidemiology of CTX-M β-lactamases has been globally changed (Bevan et al., 2017). CTX-M-55, which differs from CTX-M-15 by one nucleotide at 239 resulting in A77V substitution, displayed enhanced cephalosporin-hydrolyzing activity and structural stability (He et al., 2015). The population of CTX-M-55–producing ESBL-EC strains in China is showing increasing trends in both human and food animals (Hu et al., 2013; Rao et al., 2014; Zhang et al., 2014). In South Korea, CTX-M-15 and CTX-M-14 had been reported to be the predominant CTX-M β-lactamases in ESBL-EC isolates from food animals (Tamang et al., 2013b; Shin et al., 2017), however, CTX-M-55 was most prevalently detected in companion animals (Hong et al., 2019) and raw retail chickens (Park et al., 2019). In this study, we found that food animal ESBL-EC predominantly produced the CTX-M-55 enzyme (Table 1), suggesting that the CTX-M-55 may be supplanting CTX-M-15. The *bla*_CTX–M–55_ gene associated with cefotaxime-resistant phenotype was transferable from 14 of 24 *bla*_CTX–M–55_–positive ESBL-EC isolates to other *E. coli* strain by conjugation as described in Supplementary Table S6, suggesting that food animals may acquire *bla*_CTX–M–55_ through a A77V substitution from *bla*_CTX–M–15_ but also horizontal gene transfer. Plasmids play a critical role in the global dissemination of ESBL genes (Wang et al., 2018). The *bla*_CTX–M–55_ gene was frequently found on IncF, IncI1, and IncHI2 plasmids of *E. coli* in many countries, including China (Yang et al., 2014; Wang et al., 2018), France (Lupo et al., 2018), and United States (McGann et al., 2016). Similarly, the presence of I1-Iγ, F, and P plasmids in *bla*_CTX–M–55_–positive ESBL-EC isolates from pigs has been documented in South Korea (Tamang et al., 2013b). This study showed that most of the *bla*_CTX–M–55_–positive transconjugants (12/14, 85.7%) carried IncF replicon in combination with other types, including FIB, I1-Iγ, K, N, and/or FIA. The diversity of plasmid types was increased in comparison with those in previous report (Tamang et al., 2013b), which may reflect more influx of various antimicrobial-resistant genes. Among IncF plasmids in the *bla*_CTX–M–55_-positive *E. coli* isolates from animals in China, F33 plasmids were the most prevalent replicon STs (Yang et al., 2015). In contrast, F1 types, which are commonly found to carry ESBL genes (Brolund, 2014), were most predominantly identified in this study.

In this study, the food animal ESBL-EC isolates mainly belonged to commensal groups A or B1. Most of each phylogroup to which they belonged was allocated to a distinct cluster, whereas there was no difference between animal species allocations (Figure 2), suggesting that the phylogenetic relationships among the ESBL-EC STs may be closely related to their phylogroups regardless of the host animal species. Notably, the different breed composition of cattle has been reported to be associated with gut microbiota structure and β-lactam resistance (Fan et al., 2019), suggesting the impact of animal genetics on the antimicrobial-resistant bacteria profile even within the same species. We further detected various clonal STs in these isolates by MLST analysis. The rate of STs detected only once was 42.9%, suggesting that food animals possess a wider variety of MDR-ESBL-EC STs. Interestingly, among them, ST131, ST10, ST38, ST410, ST354, ST58, and ST117 have been reported as major extraintestinal pathogenic *E. coli* (ExPEC) lineages, which cause an extraintestinal infection in human (Manges et al., 2019). Rarely reported ST457 also belongs to ExPEC (Seni et al., 2018). The globally predominant ExPEC ST131 belongs to the highly virulent phylogroup B2 and causes both community-onset and hospital-onset infections (Nicolas-Chanoine et al., 2014). It commonly produces ESBLs and is highly associated with MDR, including resistance to fluoroquinolone. The epidemiology and characteristics of the ST131 clonal group have mostly been investigated in human clinical isolates, and animal and environmental clones have been identified only in a few studies worldwide. Similarly in South Korea, *E. coli* ST131 was found to be the most prevalent clone in patients with urinary tract infections and bacteremia and commonly harbored *bla*_CTX–M–15_ and *bla*_CTX–M–14_ (Lee et al., 2010; Cha et al., 2016; Kim H. et al., 2017, Kim Y.A. et al., 2017). There have been few reports on *E. coli* ST131 from food animals (Nicolas-Chanoine et al., 2014). To the best of our knowledge, this is the first report of the presence of *E. coli* ST131 in food animals in South Korea. We identified two pig *E. coli* ST131 isolates, which harbored both *bla*_CTX–M–65_ and *bla*_TEM–1_ genes and had MDR phenotypes. Human *E. coli* ST131 carrying both *bla*_CTX–M–65_ and *bla*_TEM–1_ has been detected in Germany (Cullik et al., 2010). Clonal populations of ST410 are present in humans, companion animals, livestock, and the environment (Falgenhauer et al., 2016) and pose a high risk of causing ExPEC outbreaks in hospitals worldwide (Roer et al., 2018). ST10, ST58, and ST117 lineages have also been detected in both humans and food animals (Manges et al., 2015; McKinnon et al., 2018). In addition, ST38 and ST101 were present in chicken and pig samples analyzed in this study, respectively, which are more related to hospital-onset than to community-onset infections in South Korea (Yoo et al., 2013; Kim et al., 2016). Extended-spectrum β-lactamase genes may circulate among food animals, farm workers, and the farm environment (Tamang et al., 2013a), but also cefotaxime-resistant bacteria can be isolated from food animals raised without cephalosporins (Mir et al., 2016, 2018), suggesting the animal acquisition of antimicrobial resistance from the environment. Together, these results suggest the two-way spread of resistant bacteria: food animals may be getting them from humans, hospital waste, and the environment or from their feed and fodder.

In conclusion, our results demonstrate the increasing occurrence and clonal diversity of MDR-ESBL-EC strains in food animals. These strains include pathogenic human-associated lineages, such as the *E. coli* ST131 clone. To explore the possible origin of the two ST131 strains found in pigs, it would be interesting to compare their core genome sequences with those of other ST131 from humans in South Korea. Given the possibility of direct transmission of antimicrobial resistance to humans through the food chain, this study also demonstrates the importance of understanding the dynamics of MDR *E. coli* in food animals.

## Data Availability Statement

All datasets generated for this study are included in the article/Supplementary Material.

## Ethics Statement

Ethical review and approval was not required for the animal study because we used livestock feces from slaughterhouses, not livestock itself.

## Author Contributions

S-SO and JSh contributed conception and design of the study. JSo, S-SO, and JK collected the samples and performed the experiments. JSh wrote the manuscript. All authors analyzed the data, contributed to manuscript revision, read and approved the submitted version.

## Conflict of Interest

The authors declare that the research was conducted in the absence of any commercial or financial relationships that could be construed as a potential conflict of interest.

## Footnotes

1 ftp://ftp.ncbi.nlm.nih.gov/pathogen/Antimicrobial_resistance

2 https://blast.ncbi.nlm.nih.gov/Blast.cgi

3 https://enterobase.warwick.ac.uk
