# Supplementary material for: Clinically Relevant Extended-Spectrum β-Lactamase–Producing Escherichia coli Isolates From Food Animals in South Korea
Source: Front Microbiol. 2020 Apr 22;11:604. doi: 10.3389/fmicb.2020.00604 (PMC7188773; doi:10.3389/fmicb.2020.00604)
Supplement: Supplementary file 1 [file Data_Sheet_1.PDF]

TABLE S1 Zone diameter breakpoints used for antimicrobial susceptibility testing in this study

Antimicrobial class	Agent	Disk content (μg)	Zone diameter breakpoints (mm) [a]			Reference
			R	I	S	
Aminoglycoside	Gentamicin	10	≤ 12	13-14	≥ 15	[1]
	Amikacin	30	≤ 14	15-16	≥ 17	[1]
Carbapenem	Ertapenem	10	≤ 18	19-21	≥ 22	[1]
	Imipenem	10	≤ 19	20-22	≥ 23	[1]
	Meropenem	10	≤ 19	20-22	≥ 23	[1]
Non-extended spectrum cephalosporin	Cefazolin	30	≤ 19	20-22	≥ 23	[1]
Extended spectrum cephalosporin	Cefotaxime	30	≤ 22	23-25	≥ 26	[1]
	Ceftazidime	30	≤ 17	18-20	≥ 21	[1]
	Cefepime	30	≤ 18	19-24	≥ 25	[1]
Cephamycin	Cefoxitin	30	≤ 14	15-17	≥ 18	[1]
Fluoroquinolone	Ciprofloxacin	5	≤ 15	16-20	≥ 21	[1]
Quinolone	Nalidixic acid	30	≤ 13	14-18	≥ 19	[1]
Folate pathway inhibitor	Trimethoprim−sulfamethoxazole	1.25/23.75	≤ 10	11-15	≥ 16	[1]
Glycylcycline	Tigecycline	15	≤ 14	15-17	≥ 18	[2]
Monobactam	Aztreonam	30	≤ 17	18-20	≥ 21	[1]
Penicillin	Ampicillin	10	≤ 13	14-16	≥ 17	[1]
	Piperacillin	100	≤ 17	18-20	≥ 21	[1]
Penicillins plus β-lactamase inhibitor	Amoxicillin−clavulanic acid	20/10	≤ 13	14-17	≥ 18	[1]
	Ampicillin−sulbactam	10/10	≤ 11	12-14	≥ 15	[1]
Phenicol	Chloramphenicol	30	≤ 12	13-17	≥ 18	[1]
Tetracycline	Tetracycline	30	≤ 11	12-14	≥ 15	[1]

[a] The antimicrobial susceptibilities were indicated as resistant (R), intermediate resistant (I), or susceptible (S).

[1] CLSI (2017). Clinical and Laboratory Standards Institute. Performance Standards for Antimicrobial Susceptibility Testing. 27th ed. CLSI supplement M100. Wayne, PA: Clinical and Laboratory Standards Institute.
[2] EUCAST (2017). The European Committee on Antimicrobial Susceptibility Testing. Breakpoint tables for interpretation of MICs and zone diameters. Version 7.1.
