# Supplementary material for: Clinically Relevant Extended-Spectrum β-Lactamase–Producing Escherichia coli Isolates From Food Animals in South Korea
Source: Front Microbiol. 2020 Apr 22;11:604. doi: 10.3389/fmicb.2020.00604 (PMC7188773; doi:10.3389/fmicb.2020.00604)
Supplement: Supplementary file 2 [file Data_Sheet_2.PDF]

TABLE S2 Primers used in this study

Target	Primer pair	Sequence (5'~3')	Annealing temperature (°C)	Size (bp)	Reference
TEM	TEM-F/R	ATGAGTATTCAACATTTCCGT/TTACCAATGCTTAATCAGTGA	59 [a]	861	[1]
SHV	SHV-F/R	CCGGGTTATTCTTATTTGTCGCT/TAGCGTTGCCAGTGCTCG	61 [b]	927	[1]
CTX-M group 1	CTX-M-1F/R	ACCGTCACGCTGTTGTTAGG/GTCGGTGACGATTTTAGCCG	56 [c]	819	[1]
CTX-M group 2	CTX-M-2F/R	AATGTTAACGGTGATGGCGA/ACCGTGGGTTACGATTTTCG	56 [c]	844	[1]
CTX-M group 9	CTX-M-9F/R	GTGCAACGGATGATGTTCG/ATGATTCTCGCCGCTGAAG	56 [c]	845	[1]
CTX-M group 25	CTX-M-25F/R	GTAAGGCGGGCGATGTTAAT/AACCGTCGGTGACAATTCTG	56 [c]	856	[1]
ChuA	ChuA-F/R	GACGAACCAACGGTCAGGAT/TGCCGCCAGTACCAAAGACA	59 [d]	279	[2]
YjaA	YjaA-F/R	TGAAGTGTCAGGAGACGCTG/ATGGAGAATGCGTTCCTCAAC	59 [d]	211	[2]
TspE4.C2	TspE4.C2-F/R	GAGTAATGTCGGGGCATTCA/CGCGCCAACAAAGTATTACG	59 [d]	152	[2]
adk	adk-F/R	ATTCTGCTTGGCGCTCCGGG/CCGTCAACTTTCGCGTATTT	53 [e]	583	[3]
fumC	fumC-F/R	TCACAGGTCGCCAGCGCTTC/GTACGCAGCGAAAAAGATTC	53 [e]	806	[3]
gyrB	gyrB-F/R	TCGGCGACACGGATGACGGC/ATCAGGCCTTCACGCGCATC	53 [e]	911	[3]
icd	icd-F/R	ATGGAAAGTAAAGTAGTTGTTCCGGCACA/GGACGCAGCAGGATCTGTT	53 [e]	878	[3]
mdh	mdh-F/R	ATGAAAGTCGCAGTCCTCGGCGCTGCTGGCGG/ TTAACGAACTCCTGCCCCAGAGCGATATCTTTCTT	53 [e]	932	[3]
purA	purA-F/R	CGCGCTGATGAAAGAGATGA/CATACGGTAAGCCACGCAGA	53 [e]	816	[3]
recA	recA-F/R	ACCTTTGTAGCTGTACCACG/AGCGTGAAGGTAAAACCTGTG	53 [e]	780	[3]
FIB	FIB-F/R	TCTGTTTATTCTTTTACTGTCCAC/CTCCCGTCGCTTCAGGGCATT	60 [f]	683	[4]

[a] PCR condition for TEM as follows: 5 min at 94°C, 35 cycles of 30 sec at 94°C, 30 sec at 59°C, 30 sec at 72°C, and a final extension step of 7 min at 72°C.
[b] PCR condition for SHV as follows: 5 min at 94°C, 35 cycles of 30 sec at 94°C, 30 sec at 61°C, 30 sec at 72°C, and a final extension step of 7 min at 72°C.
[c] PCR condition for CTX-M groups 1, 2, 9, and 25: 5 min at 94°C, 30 cycles of 30 sec at 94°C, 20 sec at 56°C, 40 sec at 72°C, and 7 min at 72°C.
[d] Multiplex PCR condition for ChuA, YjaA and TspE4.C2: 4 min at 94°C, 30 cycles of 5 sec at 94°C, 10 sec at 59°C, and 5 min at 72°C.
[e] PCR condition was as follows: 5 min at 94°C and 30 cycles of 30 sec at 94°C, 30 sec at 53°C and 30 sec 72°C, followed by 7 min at 72°. Primer sequences were obtained from the *E. coli* MLST protocol in the EnteroBase (https://enterobase.warwick.ac.uk).
[f] PCR condition for IncF containing FIB: 5 min at 94°C, followed by 30 cycles of 1 min at 94°C, 30 sec at 60°C and 1 min at 72°C, and then at 72°C for 5 min.

[1] Kim, Y.A., Lee, K., and Chung, J.E. (2018). Risk factors and molecular features of sequence type (ST) 131 extended-Spectrum-β-lactamase-producing *Escherichia coli* in community-onset female genital tract infections. *BMC Infect Dis* 18(1), 250. doi: 10.1186/s12879-018-3168-8.
[2] Clermont, O., Bonacorsi, S., and Bingen, E. (2000). Rapid and simple determination of the *Escherichia coli* phylogenetic group. *Appl Environ Microbiol* 66(10), 4555-4558. doi:10.1128/aem.66.10.4555-4558.2000.
[3] Wirth, T., Falush, D., Lan, R., Colles, F., Mensa, P., Wieler, L.H., et al. (2006). Sex and virulence in *Escherichia coli*: an evolutionary perspective. *Mol Microbiol* 60(5), 1136-1151. doi: 10.1111/j.1365-2958.2006.05172.x.
[4] Villa, L., Garcia-Fernandez, A., Fortini, D., and Carattoli, A. (2010). Replicon sequence typing of IncF plasmids carrying virulence and resistance determinants. *J Antimicrob Chemother* 65(12), 2518-2529. doi: 10.1093/jac/dkq347.
