# Supplementary material for: Clinically Relevant Extended-Spectrum β-Lactamase–Producing Escherichia coli Isolates From Food Animals in South Korea
Source: Front Microbiol. 2020 Apr 22;11:604. doi: 10.3389/fmicb.2020.00604 (PMC7188773; doi:10.3389/fmicb.2020.00604)
Supplement: Supplementary file 3 [file Data_Sheet_3.PDF]

**TABLE S3** Antimicrobial susceptibility testing of 21 antimicrobial agents from 14 classes in this study

| Antimicrobial class<br>Agent | Number (%) of antimicrobial resistant (R), intermediate resistant (I), and susceptible (S) isolates | | | | | | | | | | | |
|---|---|---|---|---|---|---|---|---|---|---|---|---|
| | Chicken (n=32) | | | Pig (n=41) | | | Cattle (n=4) | | | Total (n=77) | | |
| | R | I | S | R | I | S | R | I | S | R | I | S |
| Aminoglycoside | | | | | | | | | | | | |
| Gentamicin | 15 (46.9) | 3 (9.4) | 14 (43.8) | 20 (48.8) | 0 | 21 (51.2) | 0 | 0 | 4 (100) | 35 (45.5) | 3 (3.9) | 39 (50.6) |
| Amikacin | 0 | 0 | 32 (100) | 1 (2.4) | 0 | 40 (97.6) | 0 | 0 | 4 (100) | 1 (1.3) | 0 | 76 (98.7) |
| Carbapenem | | | | | | | | | | | | |
| Ertapenem | 0 | 0 | 32 (100) | 0 | 0 | 41 (100) | 0 | 0 | 4 (100) | 0 | 0 | 77 (100) |
| Imipenem | 0 | 0 | 32 (100) | 0 | 1 (2.4) | 40 (97.6) | 0 | 0 | 4 (100) | 0 | 1 (1.3) | 76 (98.7) |
| Meropenem | 0 | 0 | 32 (100) | 0 | 0 | 41 (100) | 0 | 0 | 4 (100) | 0 | 0 | 77 (100) |
| Non-extended-spectrum cephalosporin | | | | | | | | | | | | |
| Cefazolin | 32 (100) | 0 | 0 | 41 (100) | 0 | 0 | 4 (100) | 0 | 0 | 77 (100) | 0 | 0 |
| Extended-spectrum cephalosporin | | | | | | | | | | | | |
| Cefotaxime | 32 (100) | 0 | 0 | 41 (100) | 0 | 0 | 4 (100) | 0 | 0 | 77 (100) | 0 | 0 |
| Ceftazidime | 0 | 4 (12.5) | 28 (87.5) | 3 (7.3) | 19 (46.3) | 19 (46.3) | 0 | 2 (50.0) | 2 (50.0) | 3 (3.9) | 25 (32.5) | 49 (63.6) |
| Cefepime | 4 (12.5) | 25 (78.1) | 3 (9.4) | 11 (26.8) | 26 (63.4) | 4 (9.8) | 1 (25.0) | 3 (75.0) | 0 | 16 (20.8) | 54 (70.1) | 7 (9.1) |
| Cephamycin | | | | | | | | | | | | |
| Cefoxitin | 0 | 0 | 32 (100) | 0 | 0 | 41 (100) | 0 | 0 | 4 (100) | 0 | 0 | 77 (100) |
| Fluoroquinolone | | | | | | | | | | | | |
| Ciprofloxacin | 24 (75.0) | 2 (6.3) | 6 (18.8) | 12 (29.3) | 3 (7.3) | 26 (63.4) | 0 | 0 | 4 (100) | 36 (46.8) | 5 (6.5) | 36 (46.8) |
| Quinolone | | | | | | | | | | | | |
| Nalidixic acid | 32 (100) | 0 | 0 | 21 (51.2) | 4 (9.8) | 16 (39.0) | 0 | 0 | 4 (100) | 53 (68.8) | 4 (5.2) | 20 (26.0) |
| Folate pathway inhibitor | | | | | | | | | | | | |
| Trimethoprim-sulfamethoxazole | 16 (50.0) | 0 | 16 (50.0) | 29 (70.7) | 1 (2.4) | 11 (26.8) | 0 | 0 | 4 (100) | 45 (58.4) | 1 (1.3) | 31 (40.3) |
| Glycylcycline | | | | | | | | | | | | |
| Tigecycline | 0 | 0 | 32 (100) | 0 | 0 | 41 (100) | 0 | 0 | 4 (100) | 0 | 0 | 77 (100) |
| Monobactam | | | | | | | | | | | | |
| Aztreonam | 10 (31.3) | 6 (18.8) | 16 (50.0) | 27 (65.9) | 5 (12.2) | 9 (22.0) | 3 (75.0) | 1 (25.0) | 0 | 40 (51.9) | 12 (15.6) | 25 (32.5) |
| Penicillin | | | | | | | | | | | | |
| Ampicillin | 32 (100) | 0 | 0 | 41 (100) | 0 | 0 | 4 (100) | 0 | 0 | 77 (100) | 0 | 0 |
| Piperacillin | 32 (100) | 0 | 0 | 40 (97.6) | 1 (2.4) | 0 | 4 (100) | 0 | 0 | 76 (98.7) | 1 (1.3) | 0 |
| Penicillin plus β-lactamase inhibitor | | | | | | | | | | | | |
| Amoxicillin–clavulanic acid | 0 | 3 (9.4) | 29 (90.6) | 1 (2.4) | 13 (31.7) | 27 (65.9) | 0 | 0 | 4 (100) | 1 (1.3) | 16 (20.8) | 60 (77.9) |
| Ampicillin–sulbactam | 1 (3.1) | 9 (28.1) | 22 (68.8) | 1 (2.4) | 17 (41.5) | 23 (56.1) | 0 | 0 | 4 (100) | 2 (2.6) | 26 (33.8) | 49 (63.6) |
| Phenicol | | | | | | | | | | | | |
| Chloramphenicol | 23 (71.9) | 0 | 9 (28.1) | 32 (78.0) | 1 (2.4) | 8 (19.5) | 0 | 0 | 4 (100) | 55 (71.4) | 1 (1.3) | 21 (27.3) |
| Tetracycline | | | | | | | | | | | | |
| Tetracycline | 20 (62.5) | 1 (3.1) | 11 (34.4) | 32 (78.0) | 0 | 9 (22.0) | 0 | 0 | 4 (100) | 52 (67.5) | 1 (1.3) | 24 (31.2) |
