# Supplementary material for: Clinically Relevant Extended-Spectrum β-Lactamase–Producing Escherichia coli Isolates From Food Animals in South Korea
Source: Front Microbiol. 2020 Apr 22;11:604. doi: 10.3389/fmicb.2020.00604 (PMC7188773; doi:10.3389/fmicb.2020.00604)
Supplement: Supplementary file 4 [file Data_Sheet_4.PDF]

TABLE S4 Characteristics of 77 ESBL-EC isolates from food animals in this study.

Isolate	Origin	Province [a]	*bla* gene	Phylogroup-ST [b]	Antimicrobial agent and susceptibility [c]																				
					GEN	AMK	ETP	IPM	MEM	CFZ	CTX	CAZ	FEP	FOX	CIP	NAL	SXT	TGC	ATM	AMP	PIP	AMC	SAM	CHL	TET
EC59	Chicken	GG	CTX-M-14	A-ST48	R	S	S	S	S	R	R	S	I	S	R	R	R	S	S	R	R	S	S	R	R
EC60	Chicken	CN	CTX-M-14	A-ST93	S	S	S	S	S	R	R	S	S	S	R	R	S	S	S	R	R	S	S	R	I
EC61	Chicken	GG	CTX-M-1	D-ST354	S	S	S	S	S	R	R	S	R	S	R	R	S	S	R	R	R	S	I	S	R
EC62	Chicken	CN	CTX-M-55, TEM-1	D-ST997	S	S	S	S	S	R	R	I	R	S	R	R	S	S	R	R	R	S	S	R	R
EC63	Chicken	CN	CTX-M-65, TEM-1	B1-ST602	R	S	S	S	S	R	R	S	I	S	R	R	S	S	R	R	R	I	I	R	S
EC64	Chicken	JB	CTX-M-14	D-ST362	R	S	S	S	S	R	R	S	I	S	R	R	R	S	S	R	R	S	S	R	S
EC65	Chicken	GG	CTX-M-14, TEM-1	D-ST1011	R	S	S	S	S	R	R	S	I	S	R	R	S	S	S	R	R	S	I	R	S
EC66	Chicken	GG	CTX-M-14	D-ST362	R	S	S	S	S	R	R	S	I	S	R	R	R	S	S	R	R	S	S	R	S
EC67	Chicken	GG	CTX-M-14	A-ST93	S	S	S	S	S	R	R	S	S	S	S	R	R	S	I	R	R	S	I	R	R
EC68	Chicken	GG	CTX-M-1	D-ST354	R	S	S	S	S	R	R	S	R	S	R	R	S	S	R	R	R	S	I	S	R
EC69	Chicken	CN	CTX-M-65	D-ST5853	R	S	S	S	S	R	R	S	S	S	I	R	R	S	I	R	R	S	S	R	R
EC70	Chicken	GG	CTX-M-14, TEM-1	A-ST752	I	S	S	S	S	R	R	S	I	S	S	R	R	S	S	R	R	S	S	R	R
EC71	Chicken	GG	CTX-M-65, TEM-1	B1-ST224	R	S	S	S	S	R	R	S	I	S	R	R	S	S	R	R	R	I	I	R	S
EC72	Chicken	CB	CTX-M-1, TEM-1	A-ST6706	S	S	S	S	S	R	R	S	I	S	R	R	R	S	I	R	R	S	I	R	R
EC74	Chicken	GG	CTX-M-14	D-ST38	I	S	S	S	S	R	R	S	I	S	R	R	R	S	S	R	R	S	S	R	R
EC75	Chicken	GG	CTX-M-15	A-ST10	S	S	S	S	S	R	R	I	I	S	S	R	S	S	R	R	R	S	S	R	R
EC76	Chicken	ICN	CTX-M-14	B1-ST155	S	S	S	S	S	R	R	S	I	S	R	R	S	S	S	R	R	S	S	S	R
EC77	Chicken	CN	CTX-M-14	A-ST6850	I	S	S	S	S	R	R	S	I	S	S	R	R	S	S	R	R	S	S	R	R
EC78	Chicken	GG	CTX-M-14	B1-ST1724	R	S	S	S	S	R	R	S	I	S	S	R	R	S	S	R	R	S	S	R	R
EC79	Chicken	CN	CTX-M-14	A-ST48	R	S	S	S	S	R	R	S	I	S	R	R	R	S	S	R	R	S	S	R	S
EC80	Chicken	JB	CTX-M-14	A-ST48	R	S	S	S	S	R	R	S	I	S	R	R	R	S	S	R	R	S	S	R	S
EC81	Chicken	CN	CTX-M-65, TEM-1	B1-ST5694	R	S	S	S	S	R	R	S	I	S	R	R	S	S	I	R	R	S	I	R	R
EC82	Chicken	CN	CTX-M-1	B1-ST155	S	S	S	S	S	R	R	S	I	S	R	R	S	S	I	R	R	S	S	S	R
EC83	Chicken	GG	CTX-M-14	D-ST5853	S	S	S	S	S	R	R	S	I	S	S	R	S	S	S	R	R	S	S	S	R
EC84	Chicken	JB	CTX-M-65	A-ND	R	S	S	S	S	R	R	S	I	S	R	R	S	S	R	R	R	I	R	R	R
EC85	Chicken	GG	CTX-M-14	A-ST10	S	S	S	S	S	R	R	S	I	S	R	R	S	S	S	R	R	S	S	S	S
EC86	Chicken	ICN	CTX-M-15, CTX-M-14	A-ND	R	S	S	S	S	R	R	S	I	S	R	R	S	S	R	R	R	S	S	R	R
EC87	Chicken	GG	CTX-M-15	A-ST1290	S	S	S	S	S	R	R	I	I	S	R	R	R	S	I	R	R	S	S	S	S
EC88	Chicken	GG	CTX-M-1	A-ST6830	S	S	S	S	S	R	R	S	I	S	I	R	R	S	S	R	R	S	S	S	R
EC89	Chicken	CB	CTX-M-15, TEM-1	A-ST8622	S	S	S	S	S	R	R	I	R	S	R	R	S	S	R	R	R	S	I	S	S
EC90	Chicken	ICN	CTX-M-15, TEM-1	D-ST1140	S	S	S	S	S	R	R	S	I	S	R	R	R	S	R	R	R	S	S	R	R
EC91	Chicken	ICN	CTX-M-14, TEM-1	A-ST10	R	S	S	S	S	R	R	S	I	S	R	R	R	S	S	R	R	S	S	R	S
EC2	Pig	GG	CTX-M-55	B1-ST101	R	S	S	S	S	R	R	I	I	S	I	R	S	S	R	R	R	S	S	R	R
EC3	Pig	CN	TEM-1	B1-ST58	S	S	S	S	S	R	R	I	S	S	S	S	R	S	S	R	R	R	I	S	S
EC4	Pig	GG	CTX-M-55, TEM-1	B1-ST58	R	S	S	S	S	R	R	I	R	S	S	R	R	S	R	R	R	I	I	S	R
EC5	Pig	CN	CTX-M-55	A-ST410	R	S	S	S	S	R	R	I	R	S	R	R	R	S	R	R	R	S	S	R	R
EC7	Pig	GG	CTX-M-55	A-ST4656	S	S	S	S	S	R	R	I	I	S	R	R	S	S	R	R	R	S	S	R	R
EC8	Pig	GG	CTX-M-65, TEM-1	B1-ST58	S	S	S	S	S	R	R	S	S	S	S	S	R	S	I	R	R	I	S	R	R
EC9	Pig	CN	CTX-M-55	B1-ST101	S	S	S	S	S	R	R	I	R	S	I	R	S	S	R	R	R	S	S	S	R
EC10	Pig	ICN	CTX-M-55	A-ST542	S	S	S	S	S	R	R	S	S	S	S	S	R	S	S	R	I	S	S	R	R
EC11	Pig	GG	CTX-M-15	A-ST3171	S	S	S	S	S	R	R	S	I	S	R	R	R	S	I	R	R	S	S	R	S
EC12	Pig	CN	CTX-M-65, TEM-1	B2-ST131	S	S	S	S	S	R	R	S	I	S	S	S	R	S	R	R	R	I	I	R	R
EC13	Pig	CN	CTX-M-65, TEM-1	B2-ST131	S	S	S	S	S	R	R	S	I	S	S	S	R	S	R	R	R	S	I	R	R
EC14	Pig	GG	CTX-M-15	A-ST48	S	S	S	S	S	R	R	R	I	S	S	R	S	S	R	R	R	S	S	S	S
EC15	Pig	GG	CTX-M-55, TEM-1	A-ST10	R	S	S	S	S	R	R	I	R	S	S	S	R	S	R	R	R	I	R	R	R
EC17	Pig	CN	CTX-M-55	A-ST48	S	S	S	S	S	R	R	I	I	S	R	R	R	S	R	R	R	S	S	R	R
EC18	Pig	ICN	CTX-M-55	A-ST48	R	S	S	S	S	R	R	S	I	S	S	R	R	S	R	R	R	S	S	R	R

(*continued on next page*)

TABLE S4 (*continued*)

Isolate	Origin	Province [a]	*bla* gene	Phylogroup-ST [b]	Antimicrobial agent and susceptibility [c]																				
					GEN	AMK	ETP	IPM	MEM	CFZ	CTX	CAZ	FEP	FOX	CIP	NAL	SXT	TGC	ATM	AMP	PIP	AMC	SAM	CHL	TET
EC19	Pig	GG	CTX-M-55	A-ST2496	S	S	S	S	S	R	R	I	I	S	S	S	R	S	R	R	R	S	S	R	S
EC20	Pig	CN	CTX-M-14, TEM-1	A-ST48	S	S	S	S	S	R	R	S	I	S	R	R	R	S	S	R	R	S	I	R	R
EC21	Pig	CN	CTX-M-55, TEM-1	D-ST457	R	S	S	S	S	R	R	I	R	S	R	R	R	S	R	R	R	S	I	R	R
EC22	Pig	ICN	CTX-M-14	B1-ST1081	S	S	S	S	S	R	R	S	I	S	S	I	R	S	S	R	R	I	I	R	R
EC24	Pig	ICN	CTX-M-55, TEM-1	A-ST542	R	S	S	S	S	R	R	I	I	S	S	S	S	S	R	R	R	S	S	R	R
EC25	Pig	GG	CTX-M-15, TEM-1	A-ST1437	S	S	S	S	S	R	R	S	I	S	R	R	R	S	S	R	R	S	I	S	R
EC26	Pig	GG	CTX-M-15, TEM-1	B1-ST345	S	S	S	S	S	R	R	S	I	S	R	R	R	S	I	R	R	I	I	S	R
EC29	Pig	CN	CTX-M-55	B1-ST201	S	S	S	S	S	R	R	R	R	S	S	I	R	S	R	R	R	S	I	R	R
EC30	Pig	CN	CTX-M-55, TEM-1	B1-ST1196	S	S	S	S	S	R	R	I	R	S	R	R	R	S	R	R	R	I	I	R	R
EC31	Pig	CN	CTX-M-55	A-ST410	R	S	S	S	S	R	R	I	R	S	R	R	I	S	R	R	R	S	S	R	R
EC32	Pig	ICN	CTX-M-55	B1-ST75	R	S	S	S	S	R	R	I	I	S	S	S	R	S	R	R	R	S	S	R	R
EC33	Pig	GG	CTX-M-55, TEM-1	A-ST410	R	S	S	S	S	R	R	I	I	S	R	R	R	S	R	R	R	I	I	R	S
EC34	Pig	GG	CTX-M-15, TEM-1	A-ST1244	S	S	S	S	S	R	R	S	S	S	S	I	S	S	S	R	R	S	S	S	S
EC35	Pig	GG	CTX-M-55	B1-ST101	R	S	S	S	S	R	R	I	I	S	S	S	S	S	R	R	R	S	S	R	S
EC36	Pig	GW	CTX-M-14	A-ST2466	R	S	S	S	S	R	R	S	I	S	S	R	S	S	S	R	R	S	S	I	R
EC37	Pig	ICN	CTX-M-55, TEM-1	A-ST410	R	R	S	S	S	R	R	I	I	S	R	R	R	S	R	R	R	I	I	R	R
EC38	Pig	CN	CTX-M-15, TEM-1	B1-ST101	R	S	S	S	S	R	R	I	R	S	S	S	R	S	R	R	R	I	I	R	R
EC39	Pig	GW	CTX-M-55	B1-ST101	R	S	S	S	S	R	R	I	R	S	I	R	R	S	R	R	R	S	S	R	R
EC40	Pig	CN	CTX-M-55	B1-ST101	S	S	S	S	S	R	R	S	I	S	S	R	S	S	I	R	R	S	S	R	S
EC41	Pig	CN	CTX-M-15, TEM-1	B1-ST101	R	S	S	S	S	R	R	R	R	S	S	S	R	S	R	R	R	I	I	R	R
EC42	Pig	CN	CTX-M-15, TEM-1	A-ST10	S	S	S	S	S	R	R	S	I	S	S	S	R	S	S	R	R	S	S	R	R
EC43	Pig	GG	CTX-M-55, TEM-1	D-ST117	R	S	S	I	S	R	R	S	I	S	S	R	R	S	R	R	R	I	I	R	R
EC44	Pig	CN	CTX-M-15, TEM-1	A-ST7068	R	S	S	S	S	R	R	S	I	S	S	I	S	S	R	R	R	S	S	R	R
EC55	Pig	GG	CTX-M-14	B1-ST1246	R	S	S	S	S	R	R	S	I	S	S	S	R	S	S	R	R	S	S	R	S
EC56	Pig	CN	CTX-M-15	B1-ST847	R	S	S	S	S	R	R	S	I	S	S	S	S	S	R	R	R	S	S	S	R
EC57	Pig	CN	CTX-M-3, TEM-1	A-ST189	S	S	S	S	S	R	R	S	I	S	S	S	R	S	I	R	R	I	I	R	R
EC6	Cattle	GG	CTX-M-15	A-ST2325	S	S	S	S	S	R	R	I	I	S	S	S	S	S	I	R	R	S	S	S	S
EC16	Cattle	GG	CTX-M-55	D-ST720	S	S	S	S	S	R	R	I	R	S	S	S	S	S	R	R	R	S	S	S	S
EC27	Cattle	GG	CTX-M-65	B1-ST5728	S	S	S	S	S	R	R	S	I	S	S	S	S	S	R	R	R	S	S	S	S
EC28	Cattle	GG	CTX-M-65	B1-ST5728	S	S	S	S	S	R	R	S	I	S	S	S	S	S	R	R	R	S	S	S	S

[a] Farm location: GG, Gyeonggi; ICN, Incheon; CN, Chungnam; JB, Jeonbuk; CB, Chungbuk; GW, Gangwon.

[b] ST, *E. coli* sequence type.

[c] GEN, gentamicin; AMK, amikacin; ETP, ertapenem; IPM, imipenem; MEM, meropenem; CFZ, cefazolin; CTX, cefotaxime; CAZ, ceftazidime; FEP, cefepime; FOX, cefoxitin; CIP, ciprofloxacin; NAL, nalidixic acid; SXT, trimethoprim-sulfamethoxazole; TGC, tigecycline; ATM, aztreonam; AMP, ampicillin; PIP, piperacillin; AMC, amoxicillin-clavulanic acid; SAM, ampicillin-sulbactam; CHL, chloramphenicol; TET, tetracycline; R, resistant; I, intermediate resistant; S, susceptible.
