# Supplementary material for: Clinically Relevant Extended-Spectrum β-Lactamase–Producing Escherichia coli Isolates From Food Animals in South Korea
Source: Front Microbiol. 2020 Apr 22;11:604. doi: 10.3389/fmicb.2020.00604 (PMC7188773; doi:10.3389/fmicb.2020.00604)
Supplement: Supplementary file 5 [file Data_Sheet_5.PDF]

TABLE S5 Antimicrobial susceptibility testing of 21 antimicrobial agents from 14 classes for 77 ESBL-EC isolates

Isolate	Origin	Antimicrobial agent [a] and disk agar diffusion zone diameter (mm) [b]																				
		GEN	AMK	ETP	IPM	MEM	CFZ	CTX	CAZ	FEP	FOX	CIP	NAL	SXT	TGC	ATM	AMP	PIP	AMC	SAM	CHL	TET
EC59	Chicken	7.0	20.5	31.1	28.7	32.3	6.0	17.3	29.5	23.0	23.9	10.6	6.0	6.0	24.1	24.5	6.0	14.6	20.4	18.2	6.0	6.0
EC60	Chicken	21.6	24.0	32.8	30.6	33.0	6.0	20.3	26.1	26.3	27.1	14.3	6.0	24.4	26.0	25.3	6.0	16.1	22.4	19.7	7.0	12.5
EC61	Chicken	20.1	20.2	30.0	27.9	31.6	6.0	9.1	23.1	16.3	21.9	9.0	6.0	16.4	23.2	16.3	6.0	10.3	18.3	13.0	28.8	6.0
EC62	Chicken	19.7	21.0	30.6	27.7	31.1	6.0	6.0	17.5	17.3	23.3	9.1	6.0	21.9	24.7	11.5	6.0	8.9	20.4	16.2	6.0	6.0
EC63	Chicken	6.0	20.3	29.6	28.3	30.4	6.0	11.0	24.1	21.3	21.5	6.0	6.0	18.6	23.4	15.3	6.0	12.2	17.8	13.9	6.0	23.7
EC64	Chicken	9.6	23.2	32.2	29.2	32.6	6.0	16.7	27.8	22.6	24.5	11.8	6.0	6.0	24.8	26.1	6.0	12.5	21.9	18.6	6.0	28.0
EC65	Chicken	11.8	22.4	31.0	28.4	31.6	6.0	15.3	26.1	21.3	19.8	9.7	6.0	21.3	23.8	22.8	6.0	11.5	19.8	14.5	6.0	25.5
EC66	Chicken	10.8	22.3	31.5	30.3	32.4	6.0	16.8	28.8	22.1	24.7	11.3	6.0	6.0	25.0	27.0	6.0	11.3	22.3	18.4	6.0	27.8
EC67	Chicken	21.4	22.8	31.3	29.0	31.7	6.0	15.1	28.4	26.2	23.8	26.8	6.0	6.0	23.4	20.5	6.0	15.4	20.0	13.0	6.0	6.0
EC68	Chicken	6.0	22.0	30.0	28.5	31.2	6.0	12.8	23.6	18.0	19.5	10.0	6.0	19.0	21.0	17.0	6.0	9.6	18.5	13.8	26.3	6.0
EC69	Chicken	11.7	23.3	32.3	29.7	33.0	6.0	15.5	27.5	27.0	24.6	19.3	6.0	6.0	22.9	18.7	6.0	17.0	24.9	20.3	6.0	6.0
EC70	Chicken	13.0	22.7	32.0	30.9	33.9	6.0	17.2	27.2	24.0	23.3	21.1	6.0	6.0	23.3	25.2	6.0	12.2	20.0	16.2	6.0	6.0
EC71	Chicken	6.0	22.3	30.6	29.8	32.3	6.0	6.0	24.7	22.8	22.0	6.0	6.0	28.9	24.3	13.4	6.0	10.1	17.0	12.6	6.0	25.7
EC72	Chicken	19.3	21.2	29.8	29.6	32.1	6.0	13.4	24.7	20.0	22.2	6.0	6.0	6.0	22.6	19.8	6.0	9.6	19.0	14.0	6.0	6.0
EC74	Chicken	12.7	23.9	32.0	29.9	32.7	6.0	18.6	27.5	23.2	26.8	13.4	6.0	6.0	25.6	25.5	6.0	14.7	22.3	19.2	6.0	6.5
EC75	Chicken	20.6	22.3	30.0	30.7	31.1	6.0	6.0	19.8	20.4	19.4	24.0	6.0	23.3	24.9	14.2	6.0	11.2	21.9	18.7	6.0	6.0
EC76	Chicken	20.3	22.4	34.0	28.6	30.6	6.0	17.1	26.5	21.4	26.4	12.0	6.0	30.2	24.6	23.8	6.0	13.7	22.3	18.6	31.5	6.0
EC77	Chicken	12.3	22.6	29.5	28.9	30.8	6.0	13.6	25.2	22.2	19.1	24.2	6.0	6.0	22.4	22.3	6.0	13.4	20.3	18.1	6.0	6.0
EC78	Chicken	10.9	22.3	33.0	29.7	31.2	6.0	17.5	28.7	23.3	24.2	29.8	6.0	6.0	24.9	24.5	6.0	14.7	19.9	17.9	6.0	6.0
EC79	Chicken	8.7	21.5	31.7	29.0	31.3	6.0	18.1	27.4	23.4	23.2	11.0	6.0	6.0	23.4	24.1	6.0	12.5	19.8	17.1	6.0	25.6
EC80	Chicken	10.7	21.2	32.5	28.9	31.4	6.0	18.6	25.2	24.1	23.8	9.9	6.0	6.0	24.6	25.1	6.0	14.5	19.8	18.3	6.0	25.7
EC81	Chicken	7.8	21.8	31.8	28.6	31.8	6.0	10.0	24.6	24.1	27.2	12.3	6.0	23.7	25.0	17.1	6.0	10.9	19.9	14.9	6.0	6.0
EC82	Chicken	21.2	23.3	31.2	29.7	32.4	6.0	15.2	26.0	20.5	24.4	13.2	6.0	22.3	25.4	18.1	6.0	11.6	19.0	15.8	26.7	6.0
EC83	Chicken	21.8	23.0	30.8	29.7	32.0	6.0	15.7	28.9	23.1	24.1	25.8	6.0	21.3	22.1	24.4	6.0	13.9	20.4	17.2	28.1	6.0
EC84	Chicken	6.0	21.0	30.7	29.4	32.7	6.0	11.4	26.8	23.0	23.0	7.7	6.0	20.9	22.9	17.0	6.0	13.2	17.5	11.0	6.0	6.0
EC85	Chicken	20.3	20.8	32.6	30.7	32.3	6.0	16.2	28.9	23.3	25.0	12.3	6.0	30.4	26.3	25.0	6.0	15.0	22.7	19.5	28.5	26.0
EC86	Chicken	10.2	20.7	30.3	27.6	29.5	6.0	6.0	22.3	18.4	22.9	6.0	6.0	20.3	24.2	12.9	6.0	10.2	22.4	18.0	6.0	6.0
EC87	Chicken	20.4	21.3	28.6	29.5	30.9	6.0	11.9	20.0	21.3	23.0	6.0	6.0	6.0	25.0	17.3	7.5	12.3	22.6	20.7	28.4	23.9
EC88	Chicken	21.2	23.0	30.3	29.1	32.0	6.0	15.4	25.1	24.5	27.2	15.3	6.0	6.0	25.6	21.6	6.0	12.2	22.0	18.8	33.2	7.6
EC89	Chicken	21.8	22.9	28.4	28.6	29.9	6.0	6.0	17.3	15.1	25.2	15.0	6.0	33.7	26.4	10.3	6.0	6.0	19.6	14.0	28.3	25.8
EC90	Chicken	19.4	21.5	27.3	28.0	30.2	6.0	11.2	22.2	20.0	23.0	12.0	6.0	6.0	25.5	17.0	6.0	8.7	19.2	17.6	6.0	6.0
EC91	Chicken	9.3	21.2	31.8	28.4	30.0	6.0	16.1	27.9	22.0	22.7	12.7	6.0	6.0	25.4	23.6	6.0	6.0	19.1	15.3	6.0	24.7
EC2	Pig	7.0	19.2	29.0	27.4	30.0	7.0	7.0	19.2	20.0	26.7	19.0	6.0	19.4	22.6	12.8	6.0	9.3	20.0	18.2	6.0	7.0
EC3	Pig	19.6	20.7	29.5	27.6	30.8	15.5	21.0	19.7	29.1	23.2	23.9	19.8	7.0	23.0	26.1	6.0	14.4	9.3	13.8	29.6	26.3
EC4	Pig	7.8	21.0	27.0	27.2	28.6	7.0	7.0	18.7	15.5	25.0	24.2	6.0	7.0	21.4	11.6	6.0	7.0	17.6	14.6	27.6	6.0
EC5	Pig	8.7	22.8	31.0	26.6	30.7	7.0	7.0	19.4	17.6	22.9	7.0	6.0	7.0	21.7	12.7	6.0	10.1	19.6	18.0	6.0	6.0
EC7	Pig	19.1	20.1	32.4	27.8	31.4	7.0	11.3	19.0	19.0	22.6	9.9	6.0	18.7	23.2	15.1	6.0	11.9	20.2	17.0	6.0	6.0
EC8	Pig	21.1	23.4	29.5	29.3	33.3	7.0	11.8	26.4	25.0	24.6	35.1	23.0	7.0	23.0	17.8	6.0	12.3	17.2	16.5	6.0	7.9
EC9	Pig	19.6	21.2	29.2	26.9	29.9	7.0	7.0	18.3	16.2	21.9	20.2	6.0	29.6	21.6	13.4	6.0	10.8	18.4	16.6	28.0	6.0
EC10	Pig	21.0	21.9	31.7	28.7	29.7	7.0	17.0	26.6	25.6	23.3	32.5	24.9	7.0	24.5	21.0	6.0	18.9	22.3	22.0	6.0	6.0
EC11	Pig	21.1	22.0	27.7	28.3	31.0	7.0	9.8	21.5	20.6	20.4	7.0	6.0	7.0	22.8	18.3	6.0	14.0	20.9	19.0	6.0	22.1

(*continued on next page*)

TABLE S5 (*continued*)

Isolate	Origin	Antimicrobial agent [a] and disk agar diffusion zone diameter (mm) [b]																				
		GEN	AMK	ETP	IPM	MEM	CFZ	CTX	CAZ	FEP	FOX	CIP	NAL	SXT	TGC	ATM	AMP	PIP	AMC	SAM	CHL	TET
EC12	Pig	20.3	23.3	31.6	30.0	32.8	6.0	8.6	25.6	22.3	22.8	34.8	23.3	6.0	24.8	15.0	6.0	10.8	16.4	12.3	6.0	6.0
EC13	Pig	21.2	23.5	31.0	29.7	31.0	7.0	11.4	24.8	22.1	23.8	33.0	24.4	7.0	25.3	15.5	6.0	10.7	18.0	13.6	6.0	6.0
EC14	Pig	20.3	20.9	31.0	30.5	32.4	7.0	7.0	14.9	21.0	21.6	24.4	6.0	29.7	23.5	9.1	6.0	7.0	20.5	16.3	27.1	23.6
EC15	Pig	7.0	21.2	28.9	28.0	30.3	7.0	8.6	19.3	16.8	21.8	27.9	19.8	7.0	23.2	10.9	6.0	8.5	16.5	9.0	6.0	6.0
EC17	Pig	19.8	21.0	30.9	30.0	32.6	7.0	7.0	18.6	19.4	20.0	11.2	6.0	7.0	22.1	13.4	6.0	9.4	20.4	16.0	6.0	6.0
EC18	Pig	7.0	21.7	27.6	28.6	31.4	7.0	7.0	21.0	19.6	22.6	24.1	6.0	7.0	23.2	12.8	6.0	8.8	20.3	17.9	7.0	6.0
EC19	Pig	21.3	23.4	30.8	31.3	32.8	7.0	9.6	18.1	20.8	24.6	35.3	23.9	7.0	24.0	14.2	6.0	11.7	19.9	17.2	6.0	23.9
EC20	Pig	20.7	22.5	32.6	30.8	34.1	7.0	11.8	26.7	22.6	20.4	12.3	6.0	7.0	22.4	21	6.0	11.7	18.4	13.3	6.0	6.0
EC21	Pig	7.0	22.7	30.5	30.6	33.4	7.0	7.0	17.6	14.4	23.3	7.0	6.0	7.0	23.5	9.8	6.0	7.0	18.1	13.3	6.0	6.0
EC22	Pig	21.8	22.9	31.5	29.8	32.7	7.0	12.5	28.5	22.1	23.7	22.5	15.6	7.0	22.7	23.3	6.0	11.5	17.3	14.8	6.0	6.0
EC24	Pig	7.0	22.6	31.6	30.0	33.4	7.0	10.8	20.2	21.0	22.3	35.2	24.5	23.1	21.6	11.5	6.0	10.3	20.2	16.0	6.0	6.0
EC25	Pig	20.7	22.6	29.3	31.9	33.4	7.0	11.9	26.6	18.8	21.5	10.6	6.0	7.0	23.2	21.2	6.0	12.1	20.0	13.8	26.0	6.0
EC26	Pig	21.4	22.2	31.6	28.5	31.4	7.0	10.3	23.4	19.9	22.7	10.6	6.0	7.0	23.2	19.0	6.0	10.7	17.1	14.8	27.2	7.7
EC29	Pig	20.6	21.0	29.3	27.8	29.3	7.0	7.0	16.0	14.3	24.2	21.0	17.1	7.0	23.6	10.3	6.0	7.0	18.6	14.0	6.0	6.0
EC30	Pig	20.3	21.4	28.3	27.3	31.4	7.0	7.0	19.4	16.8	22.0	7.0	6.0	7.0	24.2	10.4	6.0	7.0	17.5	14.3	6.0	6.0
EC31	Pig	7.0	21.1	30.2	28.2	30.1	7.0	7.0	18.5	17.2	20.0	7.0	6.0	15.7	22.8	10.3	6.0	7.0	18.2	17.3	6.0	6.0
EC32	Pig	7.0	20.5	30.6	26.3	30.0	7.0	7.0	20.1	20.5	23.3	30.1	24.0	7.0	22.7	13.3	6.0	10.4	19.6	16.2	6.0	6.0
EC33	Pig	7.5	21.0	29.0	27.1	30.5	7.0	10.1	18.7	19.4	23.1	7.0	6.0	7.0	23.5	15.1	6.0	10.1	16.8	12.4	6.0	22.6
EC34	Pig	19.5	18.6	30.6	28.6	31.4	7.0	13.2	26.2	25.0	26.4	23.8	16.5	25.3	25.2	21.1	6.0	11.0	18.2	16.1	26.6	25.5
EC35	Pig	7.0	19.9	28.2	28.3	28.8	7.0	10.4	20.0	18.8	23.5	28.9	23.7	20.2	22.5	16.0	6.0	12.2	20.4	19.1	6.0	26.1
EC36	Pig	7.0	20.8	31.4	31.5	30.0	7.0	18.3	26.0	22.7	24.6	21.0	6.0	20.0	23.2	24.5	6.0	13.1	21.4	15.8	12.3	9.9
EC37	Pig	7.0	7.0	28.7	29.9	31.4	7.0	9.8	20.8	21.8	20.6	7.0	6.0	7.0	22.9	16.7	6.0	11.2	15.5	14.6	6.0	6.0
EC38	Pig	7.9	21.9	29.0	28.0	30.6	7.0	7.0	18.0	16.8	24.0	24.4	20.1	7.0	23.4	12.4	6.0	7.0	17.2	12.2	6.0	6.0
EC39	Pig	7.0	22.7	30.9	28.9	31.7	7.0	7.0	19.8	18.0	23.0	18.5	6.0	7.0	23.0	13.9	6.0	11.5	18.2	15.9	6.0	6.0
EC40	Pig	19.0	21.0	28.6	28.5	29.3	7.0	12.3	21.8	19.7	22.8	21.8	6.0	25.7	23.4	18.0	6.0	11.9	22.2	19.7	6.0	22.1
EC41	Pig	9.6	21.0	27.3	27.3	30.0	7.0	7.0	17.0	15.7	21.8	30.0	21.1	7.0	21.8	11.4	6.0	7.0	17.9	12.0	6.0	6.0
EC42	Pig	20.2	21.8	28.8	28.7	30.5	7.0	14.5	24.4	24.8	25.2	26.1	21.4	7.0	22.8	21.3	6.0	13.3	20.6	18.3	6.0	6.0
EC43	Pig	7.0	21.8	28.3	22.3	30.2	7.0	10.3	22.0	19.0	22.8	29.2	6.0	7.0	24.6	13.5	6.0	9.1	17.8	14.6	7.0	6.0
EC44	Pig	7.0	21.9	30.3	29.8	31.7	7.0	9.6	22.6	19.6	24.8	25.4	13.9	18.8	24.0	16.1	6.0	11.1	18.6	17.6	6.0	8.0
EC55	Pig	12.0	23.0	30.7	29.6	31.5	6.0	18.2	26.5	23.9	23.6	32.6	23.5	6.0	22.7	25.0	6.0	14.4	20.4	18.6	6.0	21.9
EC56	Pig	6.0	22.5	29.8	27.6	30.4	6.0	11.0	22.0	20.1	24.5	31.4	26.1	20.5	21.1	14.2	6.0	9.8	22.3	17.5	29.4	6.0
EC57	Pig	21.0	21.3	30.5	29.3	31.4	6.0	16.4	24.0	21.0	22.9	24.6	21.9	6.0	23.2	18.8	6.0	11.3	17.7	13.1	9.3	6.0
EC6	Cattle	19.7	21.8	31.3	27.5	31.3	7.0	14.6	20.9	23.3	24.4	24.4	19.7	27.8	23.3	18.4	6.0	14.1	21.2	20.3	25.9	23.8
EC16	Cattle	21.6	23.4	30.5	29.2	32.0	7.0	7.0	18.0	16.2	25.0	35.7	22.9	30.6	25.0	12.6	6.0	7.0	18.9	16.0	26.1	22.5
EC27	Cattle	20.4	21.0	28.2	26.6	30.2	7.0	12.9	24.9	22.1	21.3	29.5	22.0	26.7	24.7	15.9	6.0	14.7	21.0	19.1	23.0	22.0
EC28	Cattle	20.4	22.3	27.6	29.8	31.9	7.0	12.4	25.6	23.2	20.7	32.4	23.2	27.6	24.2	15.3	6.0	14.8	21.2	18.0	26.9	23.9

[a] Aminoglycoside (GEN, gentamicin; AMK, amikacin), carbapenem (ETP, ertapenem; IPM, imipenem; MEM, meropenem), non-extended-spectrum cephalosporin (CFZ, cefazolin), extended-spectrum cephalosporin (CTX, cefotaxime; CAZ, ceftazidime; FEP, cefepime), cephamycin (FOX, cefoxitin), fluoroquinolone (CIP, ciprofloxacin), quinolone (NAL, nalidixic acid), folate pathway inhibitor (SXT, trimethoprim-sulfamethoxazole), glycylcycline (TGC, tigecycline), monobactam (ATM, aztreonam), penicillin (AMP, ampicillin; PIP, piperacillin), penicillin plus β-lactamase inhibitor (AMC, amoxicillin-clavulanic acid; SAM, ampicillin-sulbactam), phenicol (CHL, chloramphenicol), and tetracycline (TET, tetracycline).

[b] All of the results except for those for tigecycline, which followed the EUCAST breakpoint version 7.1 (http://www.eucast.org), were interpreted according to the CLSI document M100-S27 (https://clsi.org/m100/).
