# Supplementary material for: Clinically Relevant Extended-Spectrum β-Lactamase–Producing Escherichia coli Isolates From Food Animals in South Korea
Source: Front Microbiol. 2020 Apr 22;11:604. doi: 10.3389/fmicb.2020.00604 (PMC7188773; doi:10.3389/fmicb.2020.00604)
Supplement: Supplementary file 6 [file Data_Sheet_6.PDF]

TABLE S6 Replicon types of 24 *bla*$_{CTX-M-55}$-positive ESBL-EC isolates from food animals in this study.

Isolate	Origin	Transfer (plasmid # [a])	Plasmid replicon (base pairs)																		IncF pMLST [b]
			HI1 (471)	HI2 (644)	I1-Iγ (139)	X (376)	L/M (785)	N (559)	FIA (462)	FIB (702)	W (242)	Y (765)	P (534)	FIC (262)	A/C (465)	T (750)	FIIA$_S$ (270)	F (270)	K (160)	B/O (159)	
EC2	Pig	–	–	–	–	–	–	–	–	–	–	–	–	–	–	–	–	–	–	–	–
EC4	Pig	+ (1)	–	–	+	–	–	–	–	–	–	–	–	–	–	–	–	–	+	–	–
EC5	Pig	–	–	–	–	–	–	–	–	–	–	–	–	–	–	–	–	–	–	–	–
EC7	Pig	+ (1)	–	–	–	–	–	–	–	+	–	–	–	–	–	–	–	+	+	–	F1
EC9	Pig	–	–	–	–	–	–	–	–	–	–	–	–	–	–	–	–	–	–	–	–
EC10	Pig	+ (1)	–	–	–	–	–	–	–	–	–	–	–	–	–	–	–	+	+	–	–
EC15	Pig	–	–	–	–	–	–	–	–	–	–	–	–	–	–	–	–	–	–	–	–
EC16	Cattle	+ (1)	–	–	+	–	–	–	–	–	–	–	–	–	–	–	–	–	–	–	–
EC17	Pig	+ (2)	–	–	–	–	–	+	–	+	–	–	–	–	–	–	–	+	–	–	F1
EC18	Pig	+ (1)	–	–	–	–	–	–	–	+	–	–	–	–	–	–	–	+	–	–	F20
EC19	Pig	+ (1)	–	–	–	–	–	–	–	+	–	–	–	–	–	–	–	+	–	–	F1
EC21	Pig	+ (2)	–	–	+	–	–	–	–	+	–	–	–	–	–	–	–	+	–	–	F1
EC24	Pig	–	–	–	–	–	–	–	–	–	–	–	–	–	–	–	–	–	–	–	–
EC29	Pig	–	–	–	–	–	–	–	–	–	–	–	–	–	–	–	–	–	–	–	–
EC30	Pig	–	–	–	–	–	–	–	–	–	–	–	–	–	–	–	–	–	–	–	–
EC31	Pig	+ (1)	–	–	+	–	–	–	–	+	–	–	–	–	–	–	–	+	–	–	F1
EC32	Pig	–	–	–	–	–	–	–	–	–	–	–	–	–	–	–	–	–	–	–	–
EC33	Pig	+ (1)	–	–	–	–	–	–	–	+	–	–	–	–	–	–	–	+	–	–	F1
EC35	Pig	+ (2)	–	–	–	–	–	–	–	+	–	–	–	–	–	–	–	+	–	–	F1
EC37	Pig	–	–	–	–	–	–	–	–	–	–	–	–	–	–	–	–	–	–	–	–
EC39	Pig	+ (1)	–	–	–	–	–	–	–	+	–	–	–	–	–	–	–	+	–	–	F1
EC40	Pig	+ (2)	–	–	+	–	–	–	–	+	–	–	–	–	–	–	–	+	–	–	F1
EC43	Pig	–	–	–	–	–	–	–	–	–	–	–	–	–	–	–	–	–	–	–	–
EC62	Chicken	+ (1)	–	–	–	–	–	+	+	–	–	–	–	–	–	–	–	+	–	–	ND [c]

[a] #, number.
[b] pMLST, plasmid MLST.
[c] ND, not determined.
